# Neurobiology of Schizophrenia: A Comprehensive Review

**DOI:** 10.7759/cureus.23959

**Published:** 2022-04-08

**Authors:** Enkhmaa Luvsannyam, Molly S Jain, Maria Kezia Lourdes Pormento, Hira Siddiqui, Angela Ria A Balagtas, Bernard O Emuze, Teresa Poprawski

**Affiliations:** 1 Surgery, Avalon University, School of Medicine, Willemstad, CUW; 2 Research, California Institute of Behavioral Neurosciences & Psychology, Fairfield, USA; 3 Medicine, Saint James School of Medicine, Park Ridge, USA; 4 Medicine, Ateneo de Manila School of Medicine and Public Health, Quezon City, PHL; 5 Medicine, Windsor University School of Medicine, Cayon, KNA; 6 General Medicine, Global Health Medical Center, Canlubang, PHL; 7 Emergency Medicine, St. James School of Medicine, Fort Worth, USA; 8 Psychiatry and Behavioral Sciences, St. James Medical School, Oakbrook, USA

**Keywords:** serotonin, dopamine, positive symptom, negative symptom, neurobiology, schizophrenia

## Abstract

Schizophrenia is a debilitating disease that presents with both positive and negative symptoms affecting cognition and emotions. Extensive studies have analyzed the different factors that contribute to the disorder. There is evidence of significant genetic etiology involving multiple genes such as dystrobrevin binding protein 1 (DTNBP1) and neuregulin 1 (NRG1). There is no clear link between neurotransmitter changes and the pathophysiology of schizophrenia; however, studies have shown that subcortical dopamine dysfunction is the key mechanism. Specific regions of gray and white matter changes are observed in patients with schizophrenia; gray matter changes being more significant after the onset of psychosis. These pathological changes may be implicated in the impairment of executive functioning, attention, and working memory. The disease can be managed with pharmacological treatments based on individual patient profile, patient compliance, and disease severity. The challenge of disease management sometimes persists due to the side effects. A better understanding of the pathological processes in schizophrenia may lead to more specific and effective therapies.

## Introduction and background

Schizophrenia is a chronic illness that causes psychosis with a decline in functioning. It is a multifactorial disorder affecting millions worldwide. Diagnosis of schizophrenia requires at least two or more symptoms, and at least one of the two symptoms must be a positive symptom. Positive symptoms are hallucinations, delusions, disorganized speech, and abnormal movements. Negative symptoms are flattened affect, social withdrawal, anhedonia, apathy, and lack of emotions [1]. As described in the Diagnostic and Statistical Manual of Mental Disorders, 5th edition (DSM-5), the diagnosis of schizophrenia requires presenting symptoms that cause a decline in both social and occupational functioning for at least six months [2].

Positive symptoms reflect the presence of exaggerated ideas, perceptions, or actions, whereas negative symptoms reflect the lack of normal mental functioning. The occurrence of these symptoms reflects the interplay of the neurotransmitters, especially dopamine, in the frontal, temporal, and mesostriatal brain regions [3]. The neurotransmitter release and production is the target of current medical management. Additionally, neuroanatomical changes are seen in the brain of patients with schizophrenia. These changes are seen in the prefrontal, medial, and superior temporal lobes as reduced gray matter volume [4]. MRI studies of the brain reveal structural changes in the same brain regions that are believed to affect overall functioning in patients with schizophrenia.

The pathophysiology of schizophrenia is complex, and it has been studied for years with many factors yet to be discovered. Genetic studies show that schizophrenia involves different genetic loci and is highly pleiotropic [5]. Among all neurotransmitters involved in the pathophysiology of schizophrenia, dopamine plays a major role in psychosis.

This review on the neurobiology of schizophrenia aims to explore the current studies on the genetics, neurotransmitters, and neuroanatomy involved in the disease.

## Review

Genetics

Schizophrenia has a complex mode of inheritance involving multiple genes, biological processes, and environmental factors [6]. There is a significant contribution of genetic factors to the etiology of schizophrenia. The link between multiple genes and specific DNA and protein alterations involved in the etiology of schizophrenia has not yet been identified fully [7]. However, recent large-scale genomic studies have shown specific DNA variants and how various risk alleles contribute to the disease [5].

Based on genetic studies, schizophrenia is a highly polygenic syndrome with hundreds or even thousands of distinct genetic loci involved. There are more than 100 distinct genetic loci with common alleles of various effects identified by genomic-wide association studies (GWAS). The genetic risk of the disease appears to be highly pleiotropic; for example, there are common risk variants between schizophrenia and bipolar disorder, major depressive disorder, and an autism spectrum disorder. Another genomic study shows different biological processes where genes encode a variety of synaptic proteins, such as components of the postsynaptic density (PSD) and voltage-gated calcium channel family of proteins. It also involves genes encoding glutamate receptors and dopamine receptor D2 (DRD2) with common variations. Moreover, there is a significant finding from GWAS of schizophrenia that there are multiple correlated variants in the major histocompatibility complex (MHC). These MHC variants are associated with acquired immunity, suggesting that the immune and inflammatory processes are involved in the developmental stages of schizophrenia, such as in utero, adolescence, and adulthood [5]. Components of brain development such as synapse formation, neurite outgrowth, and homeostatic plasticity are regulated by MHC class I molecules [8].

The findings from GWAS studies allow us to identify possible genes for targeted treatment and future research regarding the immune mechanisms of schizophrenia [9]. There are still a number of shortcomings, such as clarification of the pathogenesis, early diagnosis, and the treatment of schizophrenia; hence, the extent of genomics in the treatment of schizophrenia is unclear [9,10].

'Endophenotype' is an alternative approach to investigate phenotypic variation in the identification of the genes involved in schizophrenia [6]. In epidemiology, endophenotypes are quantitative biomarkers of heritable illness in the population. They are used to connect behavioral symptoms with specific phenotypes and risk genes [6]. There is extensive central nervous system involvement in the pathology of schizophrenia. These include frontal lobe changes responsible for memory and executive processes and temporal lobe changes responsible for language comprehension, auditory perception, and episodic memory [6]. These neurological disturbances in schizophrenia are predisposed by many suspected genetic loci. Studies have identified a promising association with candidate genes, including COMT, DISC1, RGS4, PPP3CC, ZDHHC8, AKT1, neuregulin, dysbindin, G72/G30, TRAR4, and alpha-7 nicotinic receptor genes [6,7]. These genes are associated with the regulation of dopamine, contributing to the underlying cause of schizophrenia. Although identifying the exact mechanism of these genetic associations is challenging, the evidence of linkage is strongest for two of these genes: dystrobrevin binding protein 1 (DTNBP1) and neuregulin 1 (NRG1). Both DTNBP1 and NRG1 are expressed at central nervous system synapses and have an impact on glutamate neurotransmission involved in schizophrenia [7].

Pathophysiology

The primary positive, negative, and cognitive symptoms of schizophrenia have been associated with many neurotransmitters, but the subcortical dopamine dysfunction remains to be the key factor in psychotic symptoms. Presynaptic dopamine dysfunction appears to mediate psychosis in schizophrenia. Stimulants such as amphetamines enhance the dopamine effect and may induce psychotic symptoms in healthy individuals. When people with schizophrenia take stimulants, they are more sensitive to psychotic effects due to increased subcortical synaptic dopamine content, dopamine synthesis, and abnormally high dopamine release following amphetamine administration. Positron emission tomography (PET) studies have shown that the increased synaptic dopamine content is localized in the striatum. In patients with schizophrenia, alterations in dopamine function within the striatum cause delusions and psychosis [3].

**Figure 1:**
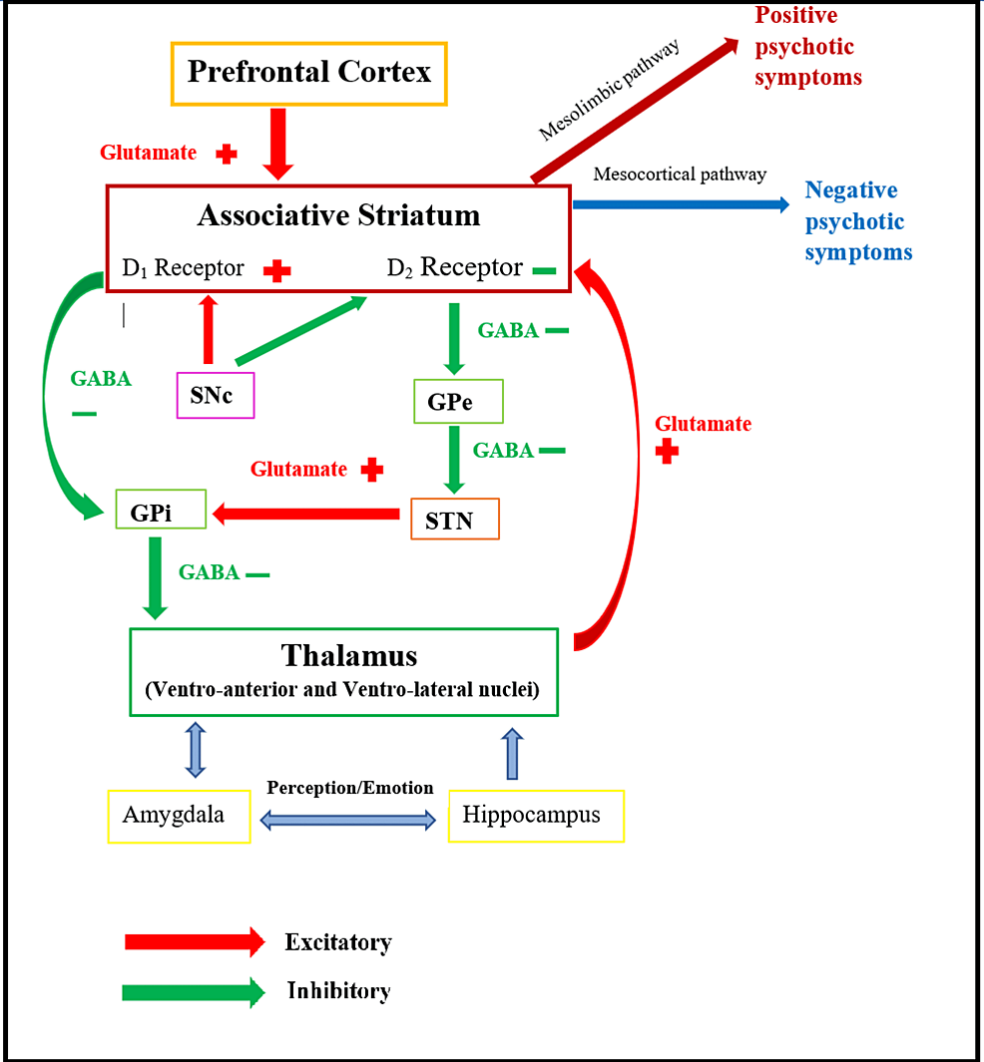
Network of direct and indirect pathways of basal ganglia involved in motor activity and psychotic symptoms; stimulation and increased activity of excessive D2 receptors in the associative striatum causing schizophrenia Original image created by the authors. D1 - dopamine D1 receptor in the excitatory pathway; D2 - dopamine D2 receptor in the inhibitory pathway; SNc - substantia nigra pars compact; GPe - globus pallidus externus; GPi - globus pallidus internus; STN - subthalamic nucleus

Thalamus is the central relay station that transmits all information from and to the cerebral cortex. The primary circuit responsible for psychotic symptoms forms between the thalamus, cerebral cortex, and associative striatum, where changes in any of these regions can impair the whole network (Figure 1). There are many more pathways involved directly or indirectly with this circuit, such as the amygdala and hippocampus, which are responsible for perception and emotion regulation. Dysfunction of the thalamus and cerebral cortex largely affects the striatum and D2 receptors, causing hallucinations and delusional symptoms [3].

Studies have shown that dopamine neurons not only release dopamine in a synaptic signal mode but also release co-transmitters glutamate and gamma-aminobutyric acid (GABA). Glutamate in the excitatory pathway and GABA in the inhibitory pathway transmits various patterns of dopamine neuron activity to the striatum. The N-methyl-D-aspartate (NMDA) receptor antagonists such as ketamine and phencyclidine (PCP) can disrupt the thalamus circuit and lead to cognitive dysfunction and psychotic symptoms [11]. Similar to amphetamine, individuals with schizophrenia are more sensitive to the effect of these medications. Hypofunction of NMDA receptors may be associated with the pathogenesis of schizophrenia; therefore, treatment with D-serine, glycine, and sarcosine, which modulates NMDA receptors, can be beneficial, especially for negative symptoms [12]. GABA interneurons such as chandelier neurons have reduced immunostaining for the GABA transporter, which is related to decreased brain-derived neurotrophic factor (BDNF) signaling or NMDA receptor hypofunction. BDNF enhances glutamatergic transmission and reduces GABAergic transmission causing alterations in neuron survival and central nervous system (CNS) function [13]. The extent to which these changes contribute to the pathophysiology of schizophrenia remains unclear.

Neuroanatomical changes

Schizophrenia is a disorder known for neuroanatomical changes over time. There are various regions of the brain that have been implicated in patients with schizophrenia. In particular, the gray matter of the brain is consistently affected in schizophrenia [14]. The widespread use of MRI has shown evidence of reduced gray matter volumes within the prefrontal, medial, and superior temporal lobes. This can explain the episodic memory decline and fluctuations in decision-making as the disease progresses [4].

Along with the respective gray matter changes, the white matter gets affected as well as schizophrenia becomes chronic [14]. Diffusion tensor imaging (DTI) is used to visualize the structural integrity of white matter using water diffusion patterns in the neural tissue. Specifically, fractional anisotropy (FA) uses the shape of the water diffusion area to assess the integrity of white matter myelination [15]. Studies on schizophrenia have shown evidence of decreased FA in major tracts such as superior longitudinal fasciculus, cingulate bundle, uncinate fasciculus, inferior longitudinal fasciculus, and hippocampus [16]. Moreover, as these pathways collaborate in neuronal networks, the increased demyelination observed in schizophrenia can also impact many cognitive abilities. A recent article proposes that the defective differentiation of glial cells is implicated in the deterioration of the working memory in schizophrenia [17]. However, a study by Dische et al. states that the brains of patients with chronic schizophrenia should be investigated carefully due to the confounding effects of antipsychotics [18]. The study confirms the decrease in the gray matter within the brain but reported less evidence of white matter abnormalities [17]. Similarly, a study by Liang et al. on the classification of schizophrenia with neuroimaging also showed significant gray matter changes compared to white matter [19].

Furthermore, CT imaging has demonstrated generalized brain tissue loss along with ventricular enlargement in schizophrenic patients as compared to controls [15]. The lateral and third ventricles progressively enlarge in size with the duration of the disease [16]. Changes associated with negative symptoms are also related to volume loss in the superior temporal lobe, medial temporal lobe, and thalamus [4]. Additionally, executive function impairment has been related to structural abnormalities in the striatum, thalamus, cerebellum, anterior cingulate gyrus, hippocampus, medial temporal lobe, medial frontal, and posterior parietal cortex [18]. Finally, it is concluded that the brain dysfunction in schizophrenic patients is due to a range of brain networks rather than a single brain region [16,18].

Neurobiology of first onset, late-onset, and relapse

A meta-analysis by Hajima et al. of medication-naive schizophrenic patients found intracranial volume decrease with chronic and recent-onset schizophrenia. The meta-analysis demonstrates that schizophrenic patients start having brain volume abnormalities in the early teenage with the first episode, which continues to a larger extent after the onset of the disease [20]. However, gray matter changes do seem more significant after the onset of psychosis and show a direct relationship with medication use and psychotic relapse [21]. Specifically, with the first episode of schizophrenia, there was evidence for both white and gray matter changes at different rates [20,21]. In both patients with or without antipsychotics, white matter changes were reflected by FA studies suggesting axonal damage or demyelination in the uncinate and arcuate fasciculi [22]. Furthermore, the most significant gray matter changes were found in the frontal and temporal lobes, including the insula, anterior cingulate gyrus, and superior temporal gyrus [23].

Late-onset schizophrenia is associated with increased ventricle-to-brain ratio, structural abnormalities in the frontal, subcortical and temporal areas, along with white matter demyelination [23]. Interestingly, late-onset schizophrenia presents with a heterogeneous set of symptoms ranging from delusions and hallucinations to cognitive impairment associated with memory declines and executive dysfunction compared to early-onset schizophrenia [24]. Studies report persecutory delusions, misidentification, and visual hallucinations as initial symptoms of late-onset schizophrenia. Other studies found an association of hallucinations representing neurocognitive disorders such as Lewy body dementia and Alzheimer's disease [25]. Memory dysfunction and cognitive abilities precede paranoid delusions. Visual and auditory hallucinations often occur concurrently with misidentification delusions, and these symptoms are associated with parietal, medial temporal, and frontal lobes dysfunction. Overall, frontotemporal abnormalities are implicated in late-life schizophrenia [24].

Schizophrenia relapse is multifactorial in nature, involving several genetic, biological, and environmental factors. It can be idiopathic or secondary to medication non-compliance or substance abuse. Clozapine seems to be an exception that has worked well for patients with relapse and treatment resistance. Overall, relapse as a whole cannot be explained by a single brain anatomical abnormality or by medication non-adherence, but it requires detailed patient history and clinical picture to account for specific reasons [26].

Neuropsychology

To understand neuropsychological impairments of schizophrenia, various psychological processes, their relativity to the area of the brain affected, and its functions need to be considered. Negative symptoms along with cognitive dysfunction are the primary reason for functional disability [27]. Even though there are various overlapping symptoms, not everyone with schizophrenia displays identical symptoms as there are distinct subtypes. This can be observed through the research conducted on paranoid versus non-paranoid schizophrenics. The patients in the paranoid group performed significantly better than those in the non-paranoid group on measures of executive functions and learning/memory [27]. When testing cognitive functions, it is evident there is impairment in working memory, verbal memory, learning, executive functions, attention, processing speed, and general intellectual disability when compared to individuals not affected by schizophrenia. Some theories state that these cognitive dysfunctions are due to impairment in connectivity between the cortices and neurotransmitter inputs [28,29].

Executive functioning is a multifaceted process where different areas of the brain function together to accomplish goal-directed behavior [30]. A study by Orellana and Slachevsky demonstrated neuroimaging with a prefrontal cortex dysfunction and further concluded that patients with schizophrenia scored relatively low on conceptualization, planning, cognitive flexibility, verbal fluency, and the ability to solve complex problems [31]. Another study explains that patients with schizophrenia exhibit reality distortion, disorganization, and psychomotor poverty, which were strongly correlated to the occupational and social impairment seen with the illness [32]. Four subtypes of attention include sustained attention, selective attention, alternating attention, and divided attention [33]. A study found that schizophrenic patients scored lower in tests such as the Stroop test for attention compared to the control group [29]. The Stroop Color-Word Test has three components: word reading, color naming, and interference (color names printed in conflicting colors), and patients with schizophrenia scored lower, possibly due to their inability to selectively focus and filter [34]. Working memory functions to gather information, code it for short or long-term storage, and apply information to attain tasks that require learning, reasoning, and comprehension [28, 35]. According to a study by Perry et al., the following five measures of working memory were administered in patients with schizophrenia: digital span forward and backward, spatial span forward and backward, and letter-number sequencing, where the patients scored significantly below average [36]. Several studies have shown severe deficits in phonological, visuospatial, and central executive areas of the working memory in schizophrenia patients. However, it is still unclear if the dysfunction is due to a specific region of the brain or the inability to synchronize the system of complex networks [28].

The neuropsychology underlying the positive and negative symptoms of schizophrenia is quite complex. The negative symptoms demonstrate a lack of initiation of emotions causing withdrawal, while positive symptoms are due to abnormal internal monitoring systems for thought and voluntary actions. The prefrontal cortex, basal ganglia, and the hippocampus are major brain regions involved in the neuropsychology of positive and negative symptoms seen in schizophrenia [37].

Pharmacology

Among many clinical trials of antipsychotic medications for schizophrenia management, the history began with first-generation antipsychotics (FGAs). The mechanism of action of the FGA is predominantly blocking the D2 receptor in the mesolimbic pathway antagonizing dopamine. Additionally, it blocks noradrenergic, cholinergic, and histaminergic actions. Moreover, first-generation antipsychotics are effective in treating positive symptoms but have no effect on negative symptoms [38]. The most common side effects of FGAs are extrapyramidal symptoms which are associated with antagonism of D2 receptors in the nigrostriatal pathway. Acute extrapyramidal symptoms include acute dystonia, akathisia, and parkinsonism, while prolonged use can lead to tardive dyskinesia [39].

Second-generation antipsychotics (SGAs) were developed for the management of both positive and negative symptoms of schizophrenia. SGAs have the added effect of antagonizing 5HT serotonin receptors along with D2 receptors causing fewer extrapyramidal symptoms in comparison to the FGAs [38]. However, there are still limitations in terms of side effects such as metabolic syndrome and hypotension. Due to metabolic syndrome, especially with olanzapine, patients with diabetes mellitus, increased BMI, and dyslipidemia should be monitored regularly [39]. Clozapine is reserved for treatment-resistant schizophrenia. It has undesirable side effects of agranulocytosis and leukopenia that may cause severe infections. The therapeutic treatment with clozapine should be halted immediately if the absolute neutrophil count drops below 1,000 cells/mm^3^ or below 500 cells/mm^3^ in those with benign neutropenia. Other possible side effects of both FGAs and SGAs include hyperprolactinemia, anhedonia, sedation, cardiac arrhythmias such as QT prolongation, and disturbances of thermoregulation. The neuroleptic malignant syndrome is a rare phenomenon that can occur within 24 to 72 hours and presents with increased temperature, severe muscular rigidity, confusion, elevation in white blood cell count (WBC), elevated creatine phosphokinase, and myoglobinuria [39].

Multiple articles report that the effect of SGAs on managing negative symptoms is debatable [40]. Clinical trials evaluating SGAs for patients with both positive and negative symptoms eventually found that the effect of these antipsychotics on negative symptoms was quite unclear. Although SGAs have more advantages for treating patients with negative symptoms compared to FGAs, it is still not the most promising treatment [40]. Lumateperone is a recently developed atypical antipsychotic that modulates serotonin, dopamine, and glutamate neurotransmission simultaneously [41]. It inhibits serotonin reuptake, antagonizes the 5-HT2A receptor and postsynaptic D2 receptor. It also acts as a partial agonist of presynaptic D2 receptors and a modulator of D1 receptor-dependent glutamate. The long-term side effects of lumateperone are currently unknown. However, it has the advantage of having fewer adverse effects due to the lack of interaction with other receptors that cause common side effects seen in most antipsychotics [41,42]. Moreover, a new drug, SEP-36385 with agonistic action on the trace amine-associated receptor 1 (TAAR1) and 5-hydroxytryptamine type 1A (5-HT1A) receptor is under investigation [43]. The SEP-363856 medication exhibited a significant decrease in psychotic symptoms in comparison to the placebo group. In a randomized controlled trial, the SEP-363856 group had gastrointestinal symptoms; however, there is no significant difference in the incidence of extrapyramidal symptoms, change in lipids, HbA1c, and prolactin level compared to the control group [43]. Therefore, longer and larger trials will be needed to confirm the potential side effects of SEP-363858 along with its efficacy in relation to the existing treatment for schizophrenia.

A new atypical antipsychotic, pimavanserin, was FDA approved in 2016 in the U.S. for Parkinson's disease psychosis (PDP), including management of hallucinations and delusions. Pimavanserin is the first drug with antipsychotic actions without dopamine D2 blocking activity. The mechanism of action of this drug involves an antagonist and an inverse agonist at the 5HT2A and 5HT2C receptors. Pimavanserin is also being studied for the management of schizophrenia, Alzheimer's disease psychosis, and major depressive disorder [44]. 

## Conclusions

Although schizophrenia is a complex syndrome that is difficult to manage, recent advances in ongoing research studies and clinical trials are contributing to the management of schizophrenia. Investigating the neurobiological processes behind behavioral disorders, including schizophrenia, will facilitate a better understanding of the pathogenesis and targeted therapy. Numerous genes have yet to be identified and may be associated with the variations in the disease, severity of cognitive impairment, and effectiveness of treatment. Further studies should integrate different domains, including genetics, biochemistry, and anatomy. A combination of all these factors will provide a deeper understanding of the pathophysiology of schizophrenia for clinicians, therefore, will contribute to the development of treatment for the illness.
